# Persistent metamorphopsia associated with branch retinal vein occlusion

**DOI:** 10.1371/journal.pone.0204015

**Published:** 2018-09-20

**Authors:** Rie Osaka, Koichiro Manabe, Saki Manabe, Yuki Nakano, Yukari Takasago, Chieko Shiragami, Kazuyuki Hirooka, Yuki Muraoka, Akitaka Tsujikawa

**Affiliations:** 1 Department of Ophthalmology, Kagawa University Faculty of Medicine, Miki, Japan; 2 Department of Ophthalmology and Visual Sciences, Kyoto University Graduate School of Medicine, Kyoto, Japan; University of Utah (Salt Lake City), UNITED STATES

## Abstract

**Purpose:**

To investigate longitudinal changes in metamorphopsia associated with branch retinal vein occlusion.

**Methods:**

In this prospective observational case series, we included 32 eyes (32 patients) with branch retinal vein occlusion and acute macular edema. Eyes were treated as needed with intravitreal ranibizumab injections for 12 months. At baseline and 1, 6, and 12 months after initiating treatment, metamorphopsia was quantified using M-CHARTS. Retinal morphology was examined through optical coherence tomography.

**Results:**

Logarithm of the minimum angle of resolution visual acuity progressively improved from 0.342 ± 0.304 (Snellen equivalent: 20/44) at baseline to 0.199 ± 0.259 (20/32) and 0.118 ± 0.195 (20/26) at 1 and 12 months, respectively (both *P* < 0.001). The M-CHARTS score significantly decreased from 0.63 ± 0.61 at baseline to 0.45 ± 0.50 at 1 month (*P* = 0.044), but no further improvement was achieved with 1 year of additional treatment (6 months: 0.47 ± 0.53 [*P =* 0.094] and 12 months: 0.50 ± 0.44 [*P =* 0.173]). Three (13.6%) of 22 eyes with baseline metamorphopsia had complete metamorphopsia resolution. At 12 months, the M-CHARTS score was correlated with baseline foveal thickness (r = 0.373, *P =* 0.035) and the baseline M-CHARTS score (r = 0.503, *P =* 0.003). A multiple regression analysis revealed that only the baseline M-CHARTS score was correlated with the 12-month M-CHARTS score (β = 0.460, *P =* 0.027).

**Conclusions:**

Eyes with branch retinal vein occlusion often have persistent metamorphopsia, even when visual acuity and retinal morphology improve. Metamorphopsia at 12 months was correlated with metamorphopsia and foveal thickness at baseline.

## Introduction

Macular edema (ME) is one of the most vision-threatening complications associated with branch retinal vein occlusion (BRVO) [1]. The visual prognosis of BRVO has substantially improved since the introduction of anti-vascular endothelial growth factor (VEGF) agents [2, 3]. In the Ranibizumab for Treatment of Macular Edema following BRVO (BRAVO) study, injections of ranibizumab (0.5 mg) were administered on an as-needed basis to patients with BRVO for 6 months. On average, visual acuity (VA) increased by 18.3 letters at 12 months [4]. However, in a clinical setting, patients with BRVO often have decreased quality of vision caused by symptomatic metamorphopsia, even after complete ME resolution.

Several investigators have used M-CHARTS to quantify metamorphopsia associated with various macular diseases [5–10]. In eyes with BRVO, Murakami and associates reported metamorphopsia in 28 (93.3%) of 30 patients with cystoid ME [11]. In our previous report on acute BRVO, 26 (89.7%) of 29 patients with metamorphopsia at baseline had persistent metamorphopsia 1 month after a single intravitreal ranibizumab injection [12]. Therefore, in patients with acute BRVO, the prevalence of metamorphopsia is high and degrades vision quality, even after treatment. However, to the best of our knowledge, no previous reports have described long-term changes in metamorphopsia associated with BRVO. It also remains unclear whether BRVO-induced symptomatic metamorphopsia regresses when ME is continuously treated. Therefore, the current study evaluated BRVO-associated metamorphopsia using M-CHARTS to determine long-term changes in metamorphopsia and identify prognostic factors.

## Patients and methods

This study was approved by the Ethics Committee at Kagawa University Faculty of Medicine (Kagawa, Japan) and was conducted in accordance with the tenets of the Declaration of Helsinki. Written informed consent was obtained from each subject before any study procedures or examinations were performed.

### Patients

This prospective study consisted of 32 consecutive patients (32 eyes) with ME associated with acute BRVO who were examined and treated in the Department of Ophthalmology at Kagawa University Hospital between October 2014 and November 2015.

The inclusion criteria of this study were (1) symptomatic BRVO with retinal hemorrhage and edema involving the macula, (2) foveal thickness greater than 250 μm at baseline (measured through optical coherence tomography [OCT]), and (3) a symptom duration of fewer than 4 months before the initial examination. The diagnosis of BRVO was determined based on fundus examinations and fluorescein angiography findings. Eyes with central retinal vein occlusion (CRVO) or hemi-CRVO were excluded from the study. Eyes with a co-morbid ocular disease known to affect OCT or angiography image quality (e.g., age-related macular degeneration, retinitis pigmentosa, diabetic retinopathy, epiretinal membrane, macular hole, retinal macroaneurysm, or senile cataract that compromised image quality) and eyes with a history of interventions for ME before study enrollment were also excluded. Some clinical information from the eligible patients was previously reported [12].

### Study examinations and treatments

At baseline, a medical history was obtained from each patient. All subjects underwent a comprehensive ophthalmological examination, including measurement of best-corrected VA using the Landolt chart, quantification of metamorphopsia with the M-CHARTS (Inami, Tokyo, Japan), indirect ophthalmoscopy, slit-lamp biomicroscopy with a noncontact lens, OCT (Spectralis HRA+OCT; Heidelberg Engineering, Heidelberg, Germany), and fluorescein angiography (Optos 200Tx imaging system, Optos PLC, Dunfermline, UK). Follow-up examinations were performed before beginning intravitreal anti-VEGF therapy (baseline, described below) and every month thereafter for a total of 12 months. At each follow-up visit, best-corrected VA was measured, and retinal morphology was examined through OCT. Fluorescein angiography was performed, if deemed necessary by the treating physician. Measurements using the M-CHARTS were performed at baseline and 1, 6, and 12 months after the initial intravitreal anti-VEGF treatments.

All subjects were treated for ME with intravitreal ranibizumab (0.5 mg Lucentis; Alcon Pharma, Tokyo, Japan). After the initial treatment, each eye was examined every month, and further injections were administered on an as-needed basis when ME or serous retinal detachment was evident at the fovea on OCT images.

### Evaluation of metamorphopsia

Metamorphopsia was quantified using commercially available M-CHARTS. The principle behind the M-CHARTS was previously described in detail [13]. Briefly, the M-CHARTS examination consists of a series of 19 dotted line tests, with dot intervals ranging from 0.2° to 2.0°. As the visual angle increases, the degree of metamorphopsia decreases. Each line has a fixation point in its center that includes 0.3° of the visual angle.

First, the examiner presents a chart with a solid line at a distance of 30 cm after correcting the refractive error with spectacles. Subsequent charts with dotted lines are then presented, with each chart having progressively increased dot spacing intervals. Subjects are asked whether the line appears distorted or straight, and when a line is perceived as straight, the visual angle of that line is taken as the degree of metamorphopsia. The M-CHARTS testing was first performed with lines in the vertical direction and then repeated with lines in the horizontal direction. Vertical and horizontal scores were independently obtained, and the higher score was used as the M-CHARTS score [12]. M-CHARTS testing was performed at baseline and 1, 6, and 12 months.

### Evaluation of retinal morphology

Retinal morphology was quantified through OCT imaging, as described previously [12]. Briefly, the entire macula was examined using sequential OCT cross-sectional images to detect serous retinal detachments and cystoid spaces. Quantitative measurements were performed on vertical sections that passed through the foveal center because the retinal portion affected by the BRVO was generally located in either the upper or lower hemisphere. In the current study, the inner retinal thickness was defined as the vertical distance between the vitreoretinal interface and outer surface of the inner nuclear layer. Outer retinal thickness was defined as the vertical distance between the outer surface of the inner nuclear layer and the inner surface of the retinal pigment epithelium (RPE). Total retinal thickness was defined as the distance between the vitreoretinal interface and inner RPE surface.

On vertical sections, inner, outer, and total retinal thicknesses were measured 1, 2, and 3 mm from the foveal center on the affected side (S1 Fig). The maximum thicknesses of the inner, outer, and total retina were defined as the maximum value among three measurements. The thickness of serous retinal detachment was manually measured at the highest detachment point, which was frequently at the fovea [14]. These measurements were obtained at baseline and 1, 6, and 12 months by one examiner who was blinded to all other clinical information.

#### Statistical analysis

Values are presented as the mean ± standard deviation, where applicable. Best-corrected VA was converted to the logarithm of the minimum angle of resolution (logMAR) for all analyses. Comparisons between time points were performed using paired Student’s *t*-tests. Bivariate relationships were examined using Pearson’s correlation coefficients or logistic regression analyses. Stepwise forward multivariate linear regression analyses were also performed to evaluate the contribution of each initially identifiable factor. Statistical analyses were performed using SPSS statistical software (version 21.0.0, IBM Japan, Tokyo, Japan), and statistical significance was defined as *P* < 0.05.

## Results

At baseline, all eyes had visual disturbances associated with acute BRVO. At this time, the mean VA was 0.342 ± 0.304 (Snellen equivalent: 20/44) and mean total foveal thickness was 504.3 ± 183.3 μm (Table 1). The maximum values of the inner, outer, and total retinal thicknesses were 309.2 ± 84.7, 316.6 ± 118.2, and 604.2 ± 163.0 μm, respectively. A total of 22 (68.8%) of 32 eyes had metamorphopsia at baseline. The mean vertical and horizontal scores were 0.55 ± 0.51 and 0.50 ± 0.62, respectively. When only the higher score from the vertical and horizontal scores was used, the mean M-CHARTS score was 0.63 ± 0.61.

**Table 1:** Baseline characteristics and treatment of eyes with acute branch retinal vein occlusion.

Number (patients/eyes)	32/32
Age (years)	68.1 ± 11.1
Sex (male/female)	15/17
Symptom duration before baseline (weeks)	10.3 ± 9.9
logMAR visual acuity	0.342 ± 0.304 (Snellen: 20/44)
Total foveal thickness (μm)	504.3 ± 183.3
Serous retinal detachment thickness (μm)	87.1 ± 100.1
Maximum total retinal thickness (μm)	604.2 ± 163.0
Maximum inner retinal thickness (μm)	309.2 ± 84.7
Maximum outer retinal thickness (μm)	316.6 ± 118.2
Number of ranibizumab injections	4.0 ± 1.8

logMAR = logarithm of the minimum angle of resolution

The mean number of ranibizumab injections administered was 4.0 ± 1.8 injections (range, 1–7 injections). No subject underwent grid laser photocoagulation or surgical intervention to treat ME during the study period. One eye of 1 subject did undergo a sub-Tenon’s injection of triamcinolone acetonide. Additionally, scatter photocoagulation was performed outside the temporal arcade vessels in 9 eyes because of non-perfused retinas. At 1 month, most eyes had a marked reduction in ME (Table 2). Both inner and outer retinal thicknesses were significantly decreased, not only at the fovea but also in the parafoveal regions (all *P* < 0.001). Additionally, VA had improved from 0.342 ± 0.304 (20/44) at baseline to 0.199 ± 0.259 (20/32, *P* < 0.001). The M-CHARTS score had decreased from 0.63 ± 0.61 at baseline to 0.45 ± 0.50 (*P* = 0.044). However, at 1 month, metamorphopsia was still present in 18 (81.8%) of 22 eyes that had metamorphopsia at baseline. The remaining 4 (18.2%) eyes had complete metamorphopsia resolution.

**Table 2:** Changes in ocular parameters following intravitreal ranibizumab therapy for branch retinal vein occlusion.

	Baseline	Month 1	Month 6	Month 12
logMAR visual acuity	0.342 ± 0.304 (Snellen: 20/44)	0.199 ± 0.259* (Snellen: 20/32)	0.126 ± 0.222* (Snellen: 20/27)	0.118 ± 0.195* (Snellen: 20/26)
Total foveal thickness (μm)	504.3 ± 183.3	261.1 ± 91.1*	217.1 ± 70.9*	228.3 ± 79.2*
Serous retinal detachment thickness (μm)	87.1 ± 100.1	5.2 ± 20.5*	1.6 ± 9.1*	7.0 ± 28.1*
Maximum total retinal thickness (μm)	604.2 ± 163.0	442.9 ± 97.6*	393.1 ± 103.3*	382.9 ± 103.3*
Maximum inner retinal thickness (μm)	309.2 ± 84.7	240.3 ± 50.6*	224.2 ± 76.2*	206.9 ± 55.3*
Maximum outer retinal thickness (μm)	316.6 ± 118.2	218.8 ± 64.4*	188.1 ± 75.5*	187.5 ± 64.1*
M-CHARTS score				
Vertical	0.55 ± 0.51	0.44 ± 0.48	0.41 ± 0.47	0.46 ± 0.43
Horizontal	0.50 ± 0.62	0.31 ± 0.42^†^	0.30 ± 0.47^†^	0.34 ± 0.39
Higher score	0.63 ± 0.61	0.45 ± 0.50^†^	0.47 ± 0.53	0.50 ± 0.44

*Paired *t*-test compared to baseline values (*P* < 0.01).

^†^Paired *t*-test compared to baseline values (*P* < 0.05).

logMAR = logarithm of the minimum angle of resolution

**Fig 1:**
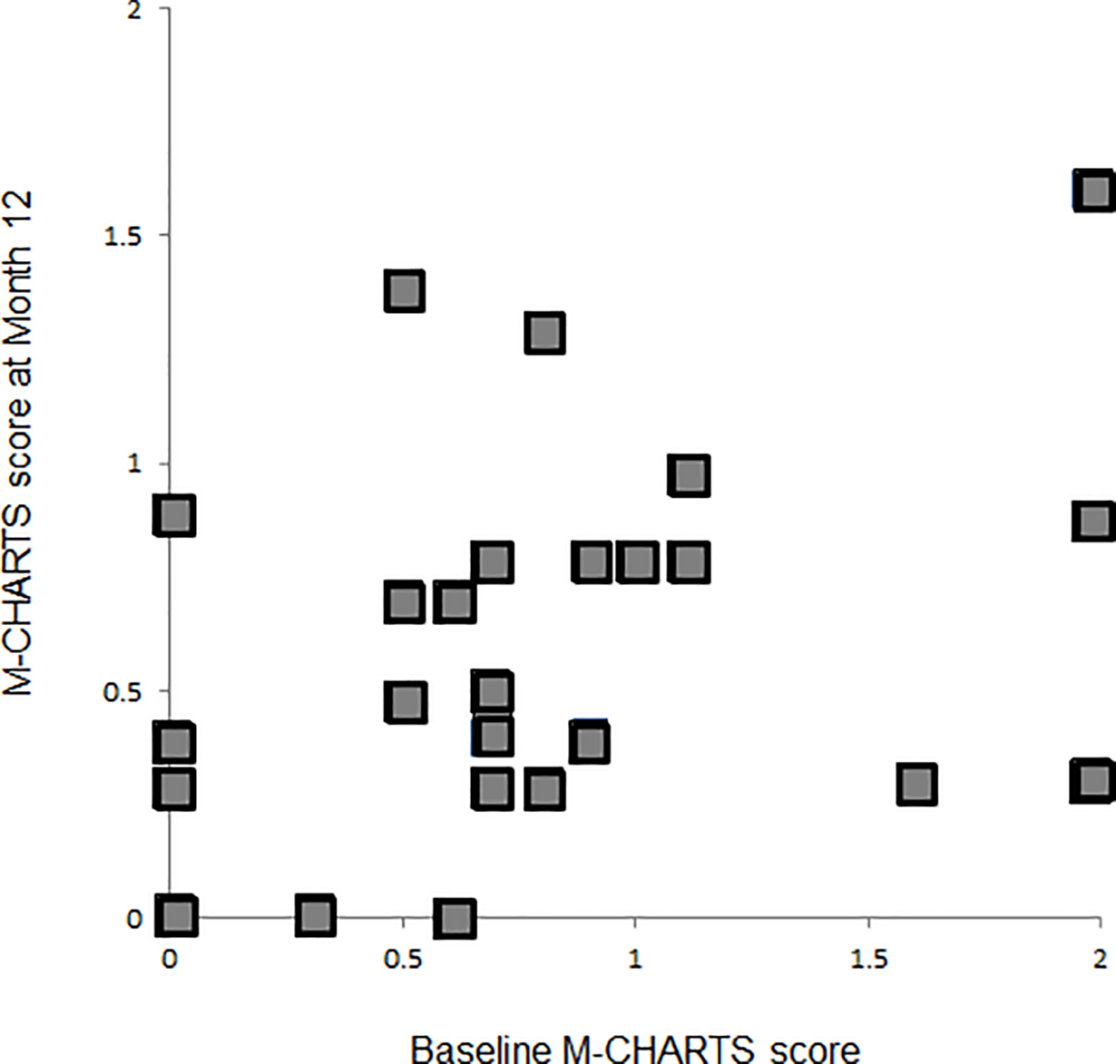
Associations between the M-CHARTS score at baseline and 12 months after initiating intravitreal ranibizumab therapy. The M-CHARTS score at baseline is significantly correlated with the M-CHARTS score 12 months after initiating intravitreal ranibizumab therapy for macular edema associated with branch retinal vein occlusion (r = 0.503, *P =* 0.003). The M-CHARTS score used in analyses was the higher score among the vertical and horizontal scores.

All included eyes maintained the reduction in ME and achieved further VA recovery during the 12-month observational period (0.118 ± 0.195 [20/26] at 12 months, *P* = 0.005 compared to 1 month; Table 2). In contrast, metamorphopsia often persisted. Of the 22 eyes that had metamorphopsia at baseline, 18 (81.8%) and 19 (86.4%) eyes still had detectable metamorphopsia at 6 and 12 months, respectively. Although the M-CHARTS score initially improved at 1 month (0.45 ± 0.50), no further improvement was achieved with 1 year of additional treatment. Rather, the scores were worsened slightly at 6 months (0.47 ± 0.53, *P =* 0.094) and 12 months (0.50 ± 0.44, *P =* 0.173; Fig 1)

**Fig 2:**
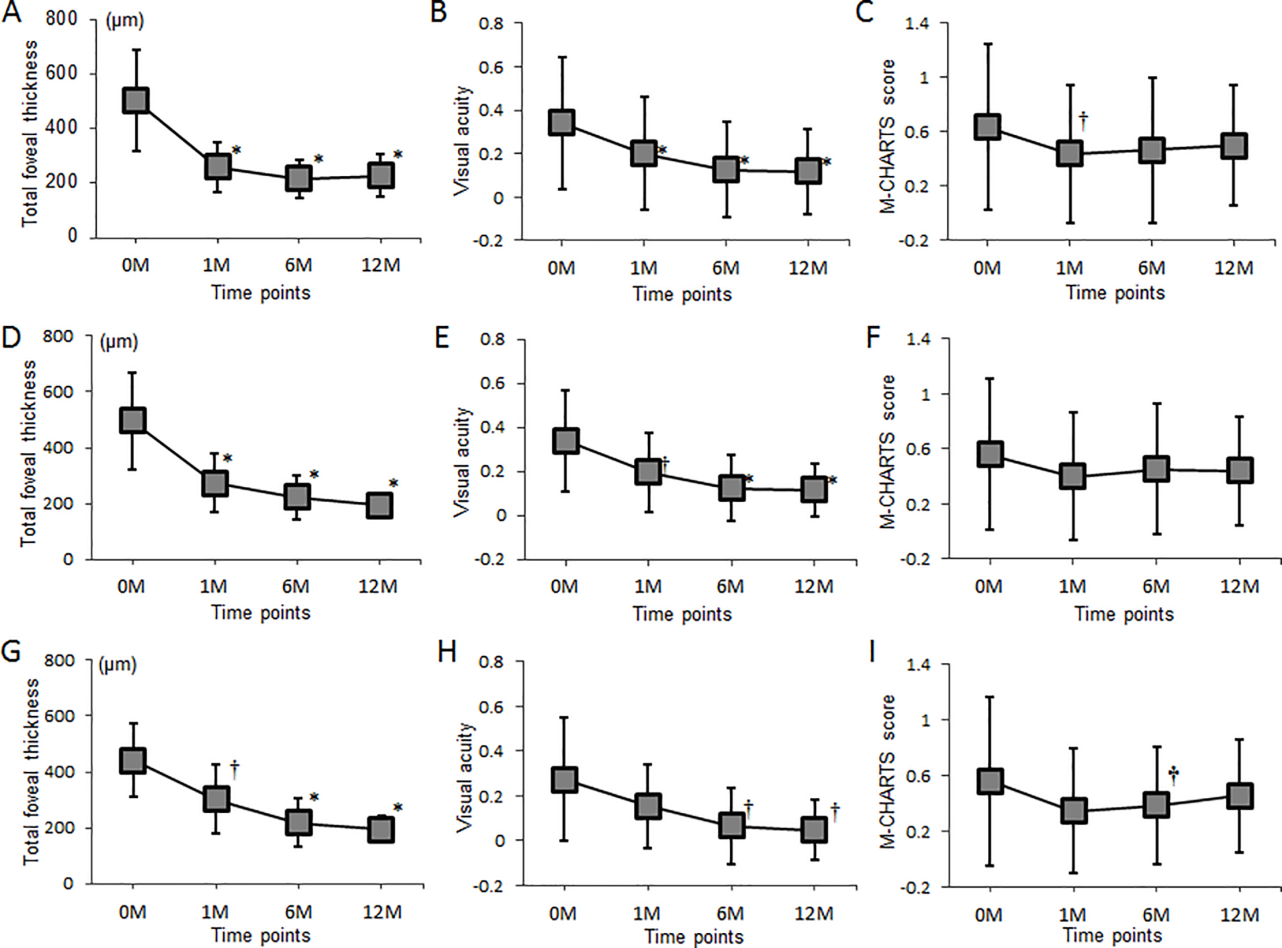
Changes in total foveal thickness, visual acuity, and M-CHARTS score after initiating intravitreal ranibizumab therapy for macular edema associated with branch retinal vein occlusion. (**A**)–(**C**), All included eyes (n = 32 eyes). (**D**)–(**F**), Eyes with no macular edema at 12 months (n = 23 eyes). (**G**)–(**I**), Eyes with an injection-free period of at least 6 months at 12 months (n = 13 eyes). Visual acuity was converted to the logarithm of the minimum angle of resolution (logMAR) for all analyses. The M-CHARTS score was the higher among the vertical and horizontal scores. *indicates *P* < 0.01 compared to baseline values, †indicates *P* < 0.05 compared to baseline values. M = month(s). Error bars represent 95% confidence intervals.

A total of 32 eyes were included in this study, and all were treated with intravitreal ranibizumab on an as-needed basis. At 12 months, 23 (71.9%) of 32 eyes had no residual ME. In the 23 eyes that had no ME at 12 months, metamorphopsia had not completely regressed although VA improvements persisted (Fig 2). Thirteen (56.5%) of these 23 eyes had injection-free periods of 6 months or longer by 12 months, but the M-CHARTS score had not improved (Fig 2).

Associations between the M-CHARTS score at 12 months and baseline measurements are summarized in Table 3. Pearson’s correlation coefficients revealed that the M-CHARTS score at 12 months was significantly correlated with the baseline total foveal thickness (r = 0.373, *P =* 0.035) and baseline M-CHARTS score (r = 0.503, *P =* 0.003). A multivariate analysis that included factors significantly correlated with the M-CHARTS score in univariate analyses revealed that only the baseline M-CHARTS score was significantly correlated with the 12-month M-CHARTS score (β = 0.460, *P =* 0.027).

**Table 3:** Association between the M-CHARTS score 12 months after initiating intravitreal ranibizumab therapy and baseline parameters in eyes with branch retinal vein occlusion.

	Bivariate correlation analysis		Multiple regression analysis			
				95% CI		
	r	*P*-value	β	Lower	Upper	*P*-value
Baseline						
LogMAR visual acuity*	0.265	0.143				
Total foveal thickness*	0.373	0.035	0.025	-0.001	0.001	0.918
Thickness of serous retinal detachment*	0.103	0.583				
Maximum total retinal thickness*	0.081	0.660				
Maximum inner retinal thickness*	0.199	0.276				
Maximum outer retinal thickness*	0.090	0.625				
Presence of serous retinal detachment^†^	-	0.626				
Presence of cystoid macula edema^†^	-	0.905				
M-CHARTS score*	0.503	0.003	0.460	0.041	0.619	0.027

The M-CHARTS score is the higher score among the vertical and horizontal M-CHARTS scores.

*Indicates that the bivariate relationship was analyzed using Pearson’s correlation coefficient.

^†^Indicates that the bivariate relationship was analyzed using a logistic regression analysis. Stepwise forward multivariate linear regression analyses were performed to evaluate the contribution of each initially identified factor. LogMAR = logarithm of the minimum angle of resolution, r = Pearson’s correlation coefficient; β = regression coefficient; CI = confidence interval

## Discussion

Several investigators have used M-CHARTS to study metamorphopsia associated with BRVO. Metamorphopsia occurs in the vast majority of eyes with BRVO, as reported by Nakagawa and associates [6] and Murakami and associates [11]. They reported a 100% (n = 12 eyes) incidence and 93.3% (n = 30 eyes) incidence, respectively. We also previously reported a 69% incidence in 42 eyes with acute BRVO [12]. In addition, metamorphopsia only resolved in 10% of acute BRVO cases 1 month after initiating intravitreal ranibizumab treatment (n = 29 eyes) [12]. Achiron and associates [5] did not observe regression of BRVO-associated metamorphopsia in any eye during a nearly 12-week treatment and follow-up period. Based on these reports, the prevalence of metamorphopsia is quite high in eyes with BRVO. Additionally, once metamorphopsia develops, it rarely regresses in the short term after treatment [5].

The mean M-CHARTS score significantly decreased from 0.63 ± 0.61 to 0.45 ± 0.50 after 1 month of intravitreal anti-VEGF therapy, but no further improvement was achieved with 1 year of additional treatment (6 months: 0.47 ± 0.53, 12 months: 0.50 ± 0.44), despite an improved VA and a decreased foveal thickness. Of the 22 eyes that had metamorphopsia at baseline, only 4 (18.2%) and 3 (13.6%) had complete metamorphopsia resolution 1 and 12 months after treatment initiation, respectively. Because eyes included in this study were being treated with intravitreal ranibizumab on an as-needed basis, we conclude that anti-VEGF therapy may initially improve metamorphopsia, but that subsequent treatments do not significantly affect it. Furthermore, complete regression of BRVO-associated metamorphopsia is rare, even with long-term, continuous treatment.

Our study subjects had recurrent or persistent ME treated with intravitreal ranibizumab on an as-needed basis. It is possible that some eyes had ME recurrence and developed subsequent metamorphopsia at 12 months. Therefore, we divided our study subjects into subgroups according to the presence or absence of ME at 12 months (Fig 2). We further analyzed that the 23 eyes without ME did not have different M-CHARTS score trends over time among all 32 eyes examined. Because sporadic ME recurrence may worsen metamorphopsia over the long term, we examined data from the 13 eyes that had an injection-free period (no ME recurrence) of longer than 6 months at 12 months. In agreement with a prior study [15], we found that VA often gradually improved after ME resolution in eyes with BRVO. However, this was not the case for BRVO-associated metamorphopsia. The only prognostic factor for persistent metamorphopsia in eyes with BRVO was the baseline M-CHARTS score (β = 0.460, *P =* 0.027). Therefore, eyes with a BRVO and higher baseline M-CHARTS score are more likely to have persistent metamorphopsia.

Metamorphopsia can make daily tasks difficult, particularly reading and writing, but mild metamorphopsia may go unnoticed under binocular vision, especially for distance vision. A previous study on eyes with epiretinal membranes reported that all patients with an M-CHARTS score of at least 0.5 were affected by metamorphopsia in their daily life [16]. Another study also concluded that an M-CHARTS score of 0.5 is the threshold for being symptomatic [7]. In the current study, 20 (62.5%) of 32 subjects and 14 (43.8%) of 32 subjects had M-CHARTS scores of at least 0.5 (symptomatic metamorphopsia) at baseline and 12 months, respectively (data not shown). A high baseline M-CHARTS score is likely a good predictor of persistent symptomatic metamorphopsia.

Various pathophysiological mechanisms have been proposed for metamorphopsia [17–19]. In acute BRVO, Murakami and associates [11] reported that the severity of metamorphopsia was correlated with the presence of inner retinal cysts in eyes with acute BRVO. Based on this, it was speculated that metamorphopsia results from horizontal, bipolar, amacrine, and Müller cell body changes caused by inner layer cysts. These changes then inhibit synaptic junctions and reduce photoreceptor sensitivity [11]. In contrast, others have theorized that metamorphopsia from acute BRVO is mainly caused by outer retinal morphological changes [12]. In macular areas affected by BRVO, macular swelling and/or serous retinal detachment would horizontally shift the retina, producing photoreceptor disorganization and subsequent metamorphopsia [12, 20]. However, multiple regression analysis showed no correlation between the M-CHARTS score at 12 months and either baseline total foveal thickness or outer retinal thickness. This may be explained by an OCT limitation that makes it fundamentally unable to detect a horizontal shift or disarray in photoreceptor cells [21].

One of the major limitations of the current study is the small sample size. As shown in Fig 2C, metamorphopsia of our patients seems to have somewhat reduced throughout the study period. The improvement was statistically significant at 1 month, but not at 6 or 12 months. This may be the result of a type 2 error caused by the small sample size. In addition, because we evaluated the metamorphopsia for 12 months after the initial treatment, the follow-up duration was rather short to study the prognosis of the visual symptoms [2]. Despite our study’s limitations, we successfully quantified BRVO-associated metamorphopsia using M-CHARTS and found that metamorphopsia often persists following intravitreal ranibizumab therapy, even after ME resolution. Additionally, a high baseline M-CHARTS score and an elevated total foveal thickness may be predictive of persistent metamorphopsia in eyes with acute BRVO.

## Supporting information

S1 Fig Optical coherence tomography measurements of retinal morphological parameters used to assess retinal changes associated with acute branch retinal vein occlusion. The vertical cross-sectional image captured through the foveal center was used. Inner (yellow arrows), outer (blue arrows), and total (red arrows) retinal thickness were measured 1, 2, and 3 mm from the foveal center on the affected side. The maximum of the inner, outer, and total retinal thickness measurements was used in analyses.
(TIF)
