# Supplementary figures and images for: Persistent metamorphopsia associated with branch retinal vein occlusion
Source: PLoS One. 2018 Sep 20;13(9):e0204015. doi: 10.1371/journal.pone.0204015 (PMC6147450; doi:10.1371/journal.pone.0204015)

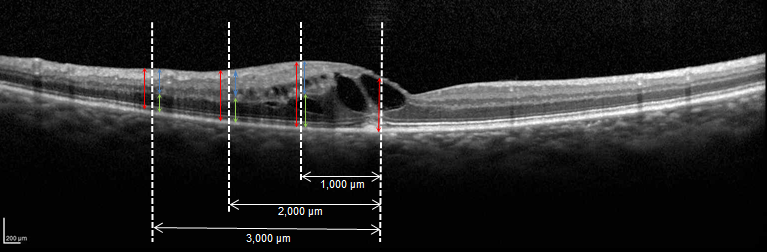

Supplement: S1 Fig — The vertical cross-sectional image captured through the foveal center was used. Inner (yellow arrows), outer (blue arrows), and total (red arrows) retinal thickness were measured 1, 2, and 3 mm from the foveal center on the affected side. The maximum of the inner, outer, and total retinal thickness measurements was used in analyses. (TIF) [file pone.0204015.s001.tif]
